# What would be a trigger tool with better performance for detecting drug-induced hyperkalemia?

**DOI:** 10.1590/1516-3180.2019.0081050719

**Published:** 2019-09-12

**Authors:** Fabiana Rossi Varallo, Rosa Camila Lucchetta, Marcela Forgerini, Patricia de Carvalho Mastroianni

**Affiliations:** I PhD. Pharmacist and Professor, Faculdade de Ciências Farmacêuticas de Ribeirão Preto (FCFRP), Universidade de São Paulo (USP), Ribeirão Preto (SP), Brazil.; II PhD. Pharmacist, Department of Pharmacy, Universidade Federal do Paraná (UFPR), Curitiba (PR), Brazil.; III BSc. Doctoral Student and Pharmacist, Department of Drugs and Medicines, School of Pharmaceutical Sciences, Universidade Estadual Paulista (UNESP), Araraquara (SP), Brazil.; IV PhD. Pharmacist and Adjunct Professor, Department of Drugs and Medicines, School of Pharmaceutical Sciences, Universidade Estadual Paulista (UNESP), Araraquara (SP), Brazil.

Dear Editor,

A trigger tool is defined as an occurrence (flag or prompt) that is easily recognized in the medical record and can alert the healthcare professional to the potential for an adverse drug event (ADE) that may not have been identified.^1^ This technique has proved to be more practical and less laborious for detecting ADEs^2^^,^^3^ than has retrospective analysis of medical records, which is more expensive and requires more time.^4^

In 2003, the Institute for Healthcare Improvement (IHI) suggested 24 trigger tools for detecting ADEs.^2^ Among these, we highlight sodium polystyrene (SPS), which is an ion-exchange resin that is used to treat hyperkalemia. It is sometimes used with sorbitol, an osmotic laxative that prevents constipation.^5^

Prescription of SPS may indicate (or “flag”) drug-induced hyperkalemia or renal impairment.^2^ Therefore, SPS can be applied as a trigger tool to detect drug-induced harm. Presence of two out of three triggers screened through use of SPS is considered to represent the existence of definite or probable ADEs, thus making SPS a trigger tool with good performance.^6^

A systematic review identified 23 therapies used in management of hyperkalemia, other than SPS.^7^ However, it was not possible to establish which therapy was the most effective and safest because of the poor quality of the studies included. In addition, there is no consensus about what serum potassium concentration is considered to represent hyperkalemia.^7^

Thus, the guidelines available for management of hyperkalemia are based on clinical experiences and practices, and on off-label use of drugs. Screening for cases of drug-induced hyperkalemia by means of serum potassium concentration can thus increase the rate of ADE reporting.

Furthermore, although SPS is standardized and available in most hospitals (given its low cost), use of SPS is questionable because of the risk of intestinal necrosis, among other serious gastrointestinal events.^7^ For this reason, use of SPS is increasingly being replaced by use of new potassium binders (patiromer and zirconium), which potentially have safer profiles than that of SPS.^8^

Hence, the physician’s choice of drug will depend on the patient’s clinical evaluation and on the safety profile of the therapies available in the healthcare services. For example, furosemide might also be a trigger for screening for drug-induced hyperkalemia since the guidelines consider it to be an option for decreasing serum potassium concentrations. Despite the off-label use, furosemide enables identification of errors or near misses. However, it cannot improve underreporting.

In this context, we consider that the serum potassium level is a more sensitive trigger tool than SPS, because using serum potassium levels as a trigger can decrease the underreporting of drug-induced hyperkalemia and increase the prevention and resolution of possible medication problems. In neonatal clinics, for instance, monitoring of serum potassium levels was found to yield performance of 100% in screening for ADEs.^9^

However, we would expect lower specificity for ADE screening in comparison with potassium binders, in the presence of clinical conditions such as renal impairment or heart failure that are considered to be confounding variables and which could lead to hyperkalemia. Similar reasoning should be applied to the serum creatinine trigger (> 1.2 mg/dl), for which specificity is only 10%, because of confounding variables such as renal failure, which decrease its performance.^6^

Therefore, further studies are needed in order to compare the performance of serum potassium levels as a trigger. Additionally, knowledge of confounding variables would enable optimization of the screening and analysis of causality.
